# Water Plasma Functionalized CNTs/MnO_2_ Composites for Supercapacitors

**DOI:** 10.1155/2013/832581

**Published:** 2013-11-19

**Authors:** Shahzad Hussain, Roger Amade, Eric Jover, Enric Bertran

**Affiliations:** FEMAN Group, IN2UB, Department de Física Aplicada i Òptica, Universitat de Barcelona, Martí i Franquès 1, 08028 Barcelona, Catalonia, Spain

## Abstract

A water plasma treatment applied to vertically-aligned multiwall carbon nanotubes (CNTs) synthesized by plasma enhanced chemical vapour deposition gives rise to surface functionalization and purification of the CNTs, along with an improvement of their electrochemical properties. Additional increase of their charge storage capability is achieved by anodic deposition of manganese dioxide lining the surface of plasma-treated nanotubes. The morphology (nanoflower, layer, or needle-like structure) and oxidation state of manganese oxide depend on the voltage window applied during charge-discharge measurements and are found to be key points for improved efficiency of capacitor devices. MnO_2_/CNTs nanocomposites exhibit an increase in their specific capacitance from 678 Fg^−1^, for untreated CNTs, up to 750 Fg^−1^, for water plasma-treated CNTs.

## 1. Introduction

Supercapacitors or electrochemical double layer capacitors are usually used in electrical circuits where high pulse power delivery is required, such as in fuel cells and hybrid electrical vehicles. Their response over extremely short time intervals and long cycle life makes them especially interesting for applications where a high specific energy and power density are needed. Depending on the charge storage mechanism, we distinguish two types of supercapacitors, electric double-layer capacitors (EDLC) and pseudocapacitors. In EDLC, the capacitance is mainly attributed to the physical adsorption of ions at the electrode/electrolyte interface as a non-Faradic behaviour, with a contribution of 1–5% pseudocapacitance in the case of carbon DLC [1], while in Faradic capacitors (pseudocapacitors), the charge storage mechanism is mainly provided by the contribution of reversible redox reactions that take place on the electrode surface involving various oxidation states of metal oxides and the physical adsorption of ions [1]. 

Several forms of carbon such as activated carbon, carbon black, and carbon nanotubes (CNTs) have been utilized in electrochemical capacitors due to their high surface area, high conductivity, thermal stability, and mechanical strength [2, 3]. Among them, vertically aligned CNTs are more suitable as electrode material because of their low contact resistance, large specific surface area, and regular pore structure in comparison with curly, random CNTs [4]. However, because of their hydrophobic nature and, therefore, low use of the specific surface area in aqueous electrolytes, the maximum capacitance obtained from pure CNTs is 40 Fg^−1^ [5]. Capacitance enhancement of carbon electrodes can be achieved by surface specific treatments that introduce various kinds of functional groups such as oxygen- and nitrogen-based functionalities. As a result, the electrodes exhibit an increase in the double layer capacitance and also of reversible faradic reactions that contribute to the charge storage as a pseudocapacitance [5, 6].

Generally, pseudocapacitance is associated with reversible Faradic redox processes in various oxidation states of transition metal oxides (RuO_2_, MnO_2_, NiO, V_2_O_5_, and IrO_2_). Among them, RuO_2_ presents the highest theoretical capacitance values (about 1400 Fg^−1^). However, due to its low cost, environmental friendliness, and, more importantly, its Faradic response with rectangular voltammograms, MnO_2_ appears to be more suitable for high power applications [7].

Regarding the oxidation/reduction mechanism in MnO_2_ pseudocapacitors, Toupin et al. suggested that H^+^ and C^+^ (Na^+^, K^+^, La^+^) are intercalated and deintercalated upon reduction and oxidation in the bulk of MnO_2_, and C^+^ is adsorbed on the surface of MnO_2_ [8]:

(1) $$\begin{array}{c} \text{Mn}{\text{O}}_{2}+{\text{H}}^{+}+{\text{e}}^{-}⟷\text{MnOOH}, \end{array}$$

or

(2) $$\begin{array}{c} \text{Mn}{\text{O}}_{2}+{\text{C}}^{+}+{\text{e}}^{-}⟷\text{MnOOC} \end{array}$$

This procedure includes a redox reaction between Mn^3+^ and Mn^4+^ oxidation states [9]. 

Since only the surface is involved in the charge storage mechanism, a thinner film of MnO_2_ shows a higher capacitance than a thicker one. However, a compromise must be achieved to obtain the optimum thickness since usually a thinner layer of manganese dioxide undergoes mechanical instability and, as a result, delivers poor energy density [10]. Therefore, for supercapacitor applications, it is important that the electrode material possesses high surface area and high porosity, as well as very low electrical resistance. A suitable configuration is CNTs/MnO_2_ composite electrodes, in which a thin layer of MnO_2_ provides high pseudocapacitance due to Faradic redox reactions taking place on large surface area electrodes. CNTs provide high electrical conductivity and mechanical stability to the 3D electrode [11, 12]. Although the highest reported capacitance obtained with such composite electrodes, in which MnO_2_ is deposited on the outer surface of CNTs, is as high as 790 Fg^−1^ [13], it is still well below the theoretical value for MnO_2_ (about 1370 Fg^−1^) [8].

Several techniques have been used to prepare MnO_2_/CNTs composite electrodes: electrophoretic deposition [14], wet chemistry method using capillary forces of the nanotubes [13], layer by layer deposition of MnO_2_ on CNTs [15], electrodeposition [12, 16, 17], and potentiostatic and galvanostatic deposition [4]. Nevertheless, there is no report in the literature about the use of water plasma-treated CNTs/MnO_2_ composite electrodes for supercapacitor applications. 

In previous work [18], we described the effect of radio frequency (rf) plasma power and water pressure on the physicochemical properties of CNTs. The specific capacitance was found to increase from 23 Fg^−1^ for untreated CNTs up to 68 Fg^−1^ for water plasma-treated carbon nanotubes (wpCNTs) with 10 W rf power and 135 Pa water pressure. Under these conditions, the water plasma is only specific to the removal of amorphous carbon, soft etching, and the introduction of oxygen functional groups. In addition, the water plasma treatment increases the wettability of the CNTs, decreases the bundle size, and increases the electrolyte accessibility to the CNTs [18]. 

Herein, we extend our approach towards the study of nanocomposite materials consisting of water plasma-treated CNTs covered with a layer of electrochemically deposited MnO_2_ (MnO_2_/wpCNTs) as electrodes for supercapacitor devices. Moreover, we study the effect of different potentials on the morphology and oxidation state of MnO_2_. 

## 2. Experimental Section

Growth and water plasma functionalization of CNTs was performed following the same procedure as previously reported [4, 18]. A Box-Wilson experimental design was adopted to explore the influence of plasma treatment parameters such as rf power and water pressure on the capacitance of MnO_2_/CNTs composite electrodes. According to the Box–Wilson graph distribution, nine conditions were chosen to perform 13 experiments; central point experiment was replicated 5 times in order to evaluate the intrinsic standard deviation of the process (see Figure S1 in Supplementary Material available at http://dx.doi.org/10.1155/2013/832581). This experimental design allows us to adjust the tested variables with a second degree polynomial equation:

(3) $$\begin{array}{c} X=\alpha_{0}+\alpha_{1}A+\alpha_{2}B+\alpha_{3}A^{2}+\alpha_{4}AB+\alpha_{5}B^{2}, \end{array}$$

where *X* is the dependent magnitude, *α*_*n*_ are constant coefficients obtained by statistically adjusting these polynomial equations, *A* is the plasma power, and *B* the water pressure.

The galvanostatic method was adopted to electrochemically deposit manganese dioxide on the surface of CNTs. A detailed description of this procedure can be found elsewhere [4]. In brief, a graphite electrode was used as a cathode and CNTs as the anode in a two-electrode cell configuration [19]. About 0.5 mL of a 0.2 M MnSO_4_ *·*H_2_O solution was introduced dropwise through a hole in the cathode during the deposition. The deposition time was set to 2 minutes at a constant current density of 1 mA cm^−2^. The amount of manganese dioxide deposited on the CNTs was determined by oxidizing the manganese ions to MnO^4-^ and measuring its absorbency at 525 nm. The concentration of permanganate ions was obtained from UV-vis spectrophotometry, and the deposited mass of MnO_2_ was calculated from this value, which equaled (16.5 ± 1.5) *μ*g. This amount corresponds to about 23% of the total mass of MnO_2_/CNTs nanocomposite. The charge storage mechanism of MnO_2_/CNTs composite electrodes was studied by means of cyclic voltammetry (CV), electrochemical impedance spectroscopy (EIS), and constant current charge/discharge cycling in a 0.1 M Na_2_SO_4_ aqueous solution using a potentiostat/galvanostat (AutoLab, PGSTAT30, USA). All experiments were carried out in a typical three-electrode cell at 25°C. A Ag/AgCl electrode (3 M KCl internal solution) and a Pt-ring electrode were used as the reference and counter electrode, respectively. The working electrode was a sample of CNTs or MnO_2_/CNTs composite. The geometrical area of the working electrode was set to a constant value of 0.57 cm^2^.

XPS experiments were performed in a PHI 5500 Multitechnique System (from Physical Electronics) with a monochromatic X-ray source (Aluminium Kalfa line of 1486.6 eV energy and 350 W), placed perpendicular to the analyzer axis, and calibrated using the 3d5/2 line of Ag with a full width at half maximum (FWHM) of 0.8 eV. The analyzed area was a circle of 0.8 mm diameter, and the selected resolution for the spectra was 187.5 eV of pass energy and 0.8 eV/step for the general spectra and 11.75 eV of pass energy and 0.05 eV/step for the spectra of the different elements in the depth profile spectra. All measurements were made in an ultra high vacuum (UHV) chamber pressure between 5 × 10^−9^ and 2 × 10^−8^ torr. Raman analysis was performed by using Raman system, HORIBA LabRam HR800 Japan. A green laser of wavelength 532 nm and power 0.5 mW and a 100x objective was used during the measurements.

## 3. Results and Discussion

**Figure 1:**
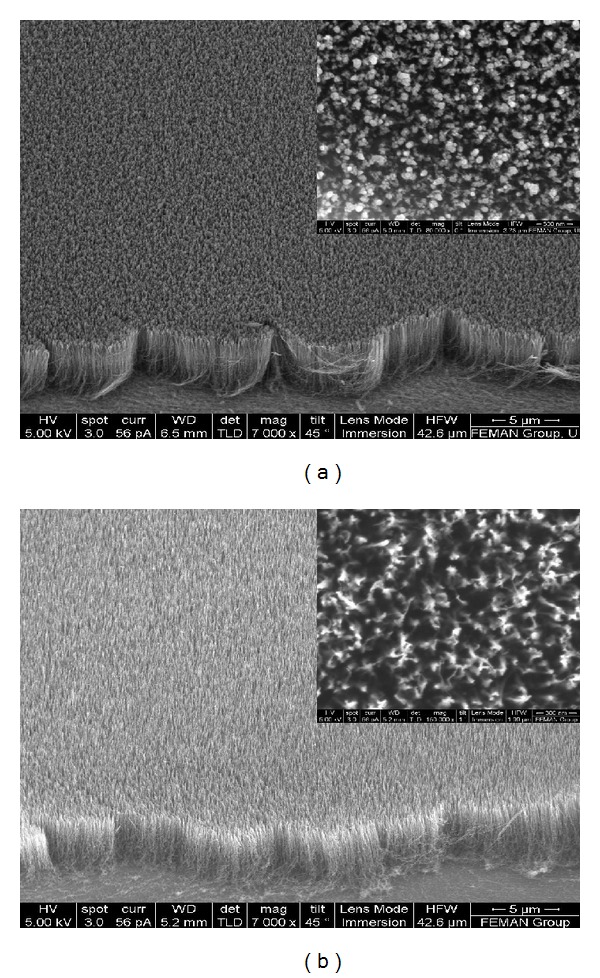
SEM images of untreated CNTs (a) and wpCNTs (b). Inset graphs show top view of the CNTs mat.

**Figure 2:**
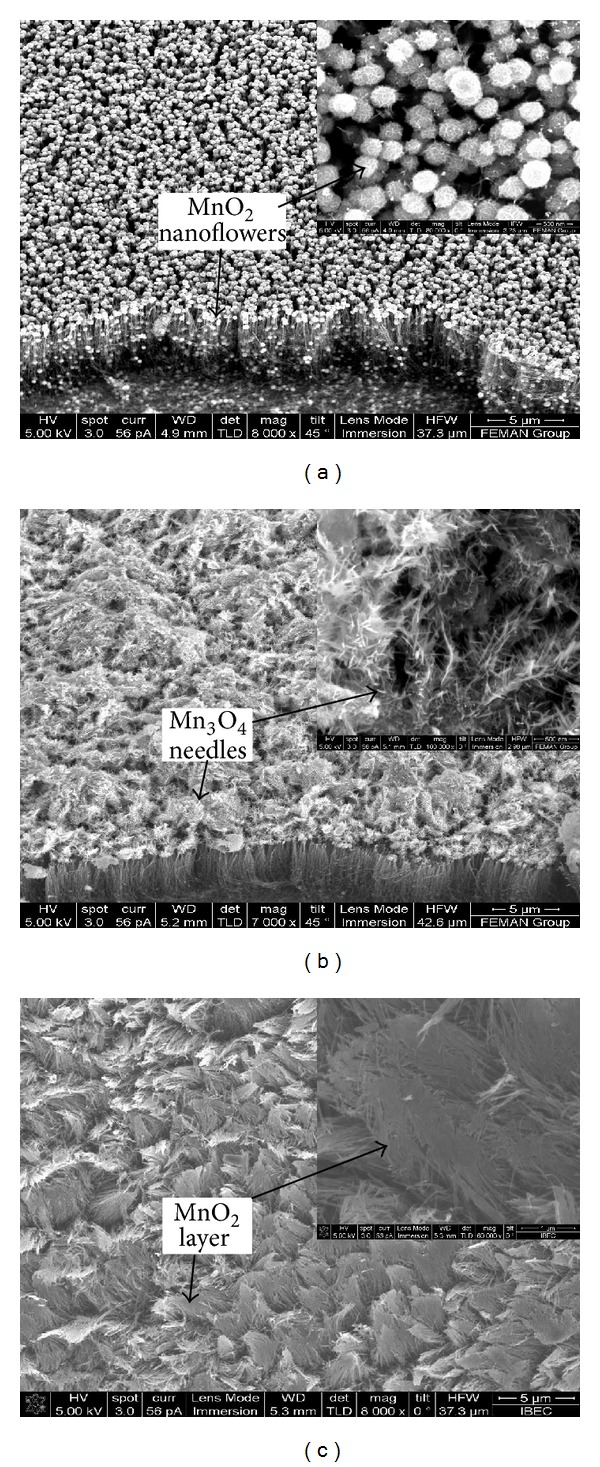
SEM images of wpCNTs/MnO_2_ before electrochemical cycling (a), wpCNTs/MnO_2_ after electrochemical cycling (0-1 V) (b), and wpCNTs/MnO_2_ after electrochemical cycling (0.1–0.8 V) (c). Inset graphs show top view of the CNTs mat.

The morphological analysis of the CNTs was performed by field emission scanning electron microscopy (FE-SEM) (FEI Nova NanoSEM 230, USA). Figures 1(a) and 1(b) show SEM images of untreated CNTs and water plasma-treated CNTs (wpCNTs) at 75 W rf power and 135 Pa pressure, respectively. The CNTs vertical alignment is preserved after all the treatments. Clearly, the CNTs surface becomes cleaner after the water plasma-treatment and presents less amorphous carbon, which is a by-product generated during the CNTs growth (inset of Figures 1(a) and 1(b)). Figures 2(a) and 2(b) show scanning electron micrographs of wpCNTs/MnO_2_ composite before and after 2000 charge/discharge cycles, respectively, in the potential range of 0-1 V. Before cycling, the MnO_2_ presents nanoflower or nanosphere-like structures that are well distributed on the CNTs tips and inside the voids of CNTs bundles reaching the bottom part of the CNTs mat (see Figure 2(a)). After 2000 cycles and within the potential window of 0 to 1 V, the morphology of MnO_2_ has changed from nanoflower to needle-like structure, which corresponds to Mn_3_O_4_ as indicated by X-ray photoelectron spectroscopy (XPS) and Raman spectroscopy (see further below). Furthermore, the Mn_3_O_4_ has agglomerated on the top surface of the CNTs mat creating a thick layer of the oxide (Figure 2(b)), whereas after 2000 charge/discharge cycles in a smaller potential window (0.1–0.8 V), nanoflower-structured MnO_2_ is transformed into a thin layer-like structure, resembling a paste coating the surface of the CNTs (see Figure 2(c)).

Structural and chemical information about the carbon nanotubes and their composites was provided by Raman spectroscopy measurements. Information regarding the CNTs structure is usually obtained from three Raman peaks that appear in the region between 1000 cm^−1^ and 1800 cm^−1^. After the deposition of manganese dioxide on untreated and water plasma-treated CNTs, new Raman peaks emerge in the frequency region from 200 to 650 cm^−1^. The peaks observed in the 200–500 cm^−1^ region are assigned to distortion of Mn–O–Mn chains in MnO_2_ octahedral lattices, while peaks in the 500–650 cm^−1^ region are characteristic of Mn–O vibration in the basal plane of MnO_6_ groups [20]. MnO_2_ Raman spectrum consists of three main distinguished peaks at 523, 576, and 650 cm^−1^ and three smaller peaks, not always detected, at 392, 490, and 776 cm^−1^ [21].

**Figure 3:**
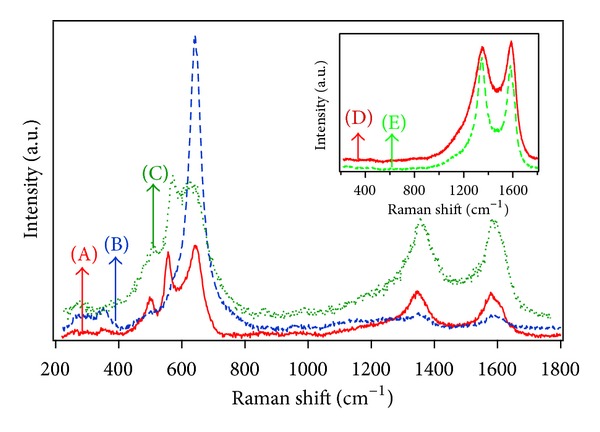
Raman spectra of (A) wpCNTs/MnO_2_ (75 W, 135 Pa) before cycling, (B) wpCNTs/MnO_2_ (75 W, 135 Pa) after 2000 cycles (between 0-1 V), and (C) wpCNTs/MnO_2_ (75 W, 135 Pa) after 2000 cycles (between 0.1–0.8 V). Inset figure shows Raman spectra of (D) untreated CNTs and (E) wpCNTs (75 W, 135 Pa).

Figure 3 shows Raman spectra of untreated CNTs, wpCNTs (75 W, 135 Pa), and wpCNTs/MnO_2_ (75 W, 135 Pa) composite before and after electrochemical cycling in small and large voltage windows. Before cycling, the peaks at 490, 560, and 640 cm^−1^ are very well pronounced and their positions are similar to those described in the literature for MnO_2_ Raman spectra, as well as the two small peaks observed below 400 cm^−1^ [21, 22]. After 2000 electrochemical charge/discharge cycles in the range of 0 to 1 V, a very sharp and high intensity peak is observed at 640 cm^−1^ along with a combination of smaller peaks at 260, 350, and 480 cm^−1^.

The high intensity of the Raman peak at 640 cm^−1^ [22] is the signature of the Mn_3_O_4_ phase and characteristic of all needle-like structures, while a low intensity peak at this frequency cannot be assigned to Mn_3_O_4_ [21, 23]. In view of these results, we assume that dissolution of manganese species from the electrode in the solution and a slow phase transformation process from MnO_2_ to Mn_3_O_4_ take place during constant current charge/discharge cycling owing to irreversible redox reactions that occur in the region around 0 V [24]. In agreement with this assumption, Gao et al. reported the thermal transformation of MnOOH into Mn_3_O_4_ phase [25]. Moreover, the above results are consistent with the morphological changes observed with SEM (see Figures 2(a) and 2(b)) since the Raman signals at ~1360 and ~1580 cm^−1^ are very weak due to the formation of a thick layer of Mn_3_O_4_ at the CNTs top surface.

Raman spectra of samples cycled in the potential range of 0.1–0.8 V present peak positions and intensities that cannot be attributed to a change in the oxidation state of MnO_2_. Therefore, only a morphological change from nanoflower to layer-like structure, as proved by SEM images, occurs after cycling in this voltage window (0.1–0.8 V). 

**Figure 4:**
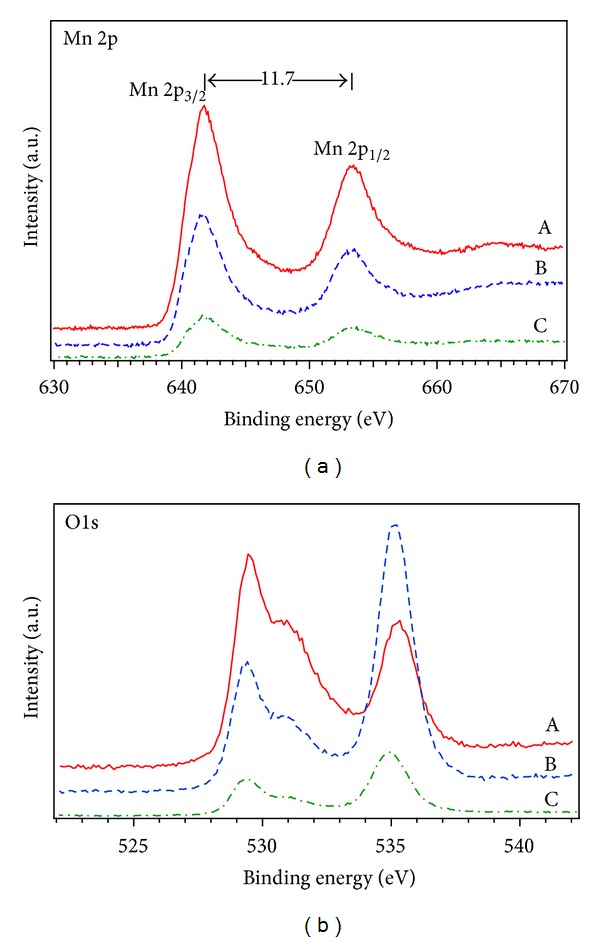
XPS spectra of Mn 2p and O1s (a) wpCNTs/MnO_2_ (75 W, 135 Pa) before cycling (b) wpCNTs/MnO_2_ (75 W, 135 Pa) after 2000 cycles (between 0-1 V), and (c) wpCNTs/MnO_2_ (75 W, 135 Pa) after 2000 cycles (between 0.1–0.8 V).

XPS was used to evaluate the elemental composition and oxidation states of manganese before and after electrochemical charge-discharge cycling within different potential limits.  Figure 4 shows Mn 2p spectra of different samples. The Mn 2p spectrum of sample wpCNTs/MnO_2_ (75 W, 135 Pa) before cycling exhibits three peaks; Mn 2p_3/2_ was positioned at (641.85 ± 0.1) eV, Mn 2p_1/2_ at (653.5 ± 0.1) eV [26], and a shoulder appears at around 644.5 eV. The binding energy difference between the Mn 2p_3/2_ and Mn 2p_1/2_ peaks is about 11.7 eV which is very much like the reported values in other studies [27]. These results indicate that manganese is in the Mn^4+^ oxidation state [28]. After 2000 charge-discharge cycles in the potential range of 0-1 V, the binding energy of the Mn 2p_3/2_ peak is shifted downward to a lower valence state of Mn at (641.5 ± 0.1) eV (see Figure 4(b)). This peak position reveals the transformation of MnO_2_ into Mn_3_O_4_ due to the irreversible potential limits used during the cycling process [28], whereas when the cycling was performed in the potential range of 0.1–0.8 V, there was almost negligible shift of the binding energies of the Mn 2p spectra in comparison with the sample without cycling, confirming that manganese is still in the Mn^4+^ state. The decrease observed in the intensities of the peaks could be related to the transition of MnO_2_ from flower-like to (sheet) layer-like structure on the nanotubes surface (see Figure 2(c)).

The chemical environment of the oxygen atoms was examined by deconvoluting the O1s spectra in 4 peaks (see Figure 4(b)). The peak located at (529.30 ± 0.1) eV can be ascribed to (Mn–O–Mn). Peaks around (530.9 ± 0.1) eV, (532.7 ± 0.2) eV, and (535.10 ± 0.2) eV could be attributed to (Mn–O–H), (H–O–H), and chemisorbed oxygen, respectively [29, 30]. Table S4 in supplementary material shows the deconvoluted O1s spectra peak position and relative area percentage of oxide species. The amount of the OH/H_2_O is higher in the sample without cycling. Thus, higher hydroxide content implies higher effective area as well as increased Na^+^ and H^+^ diffusion in the electrode [31]. The above XPS measurements are also in accordance with both SEM and Raman results previously discussed.

Specific capacitance of the wpCNTs/MnO_2_ composite electrodes was calculated using (4) as follows:

(4) $$\begin{array}{c} C_{\text{s}}=\frac{q_{a}+\left|q_{c}\right|}{2m\Delta V}, \end{array}$$

where *q*_*a*_ and *q*_*c*_ are the anodic and cathodic charges, respectively, in C, *C*_*s*_ is the specific capacitance in Fg^−1^, *m* is the mass of the active material in g, and ∆*V* the voltage in V.  Table 1 gives specific capacitance values obtained for different samples at a scan rate of 10 mVs^−1^. The specific capacitance of untreated CNTs/MnO_2_ composite is 678 Fg^−1^ and increases for most of the wpCNTs/MnO_2_ composites.

**Figure 5:**
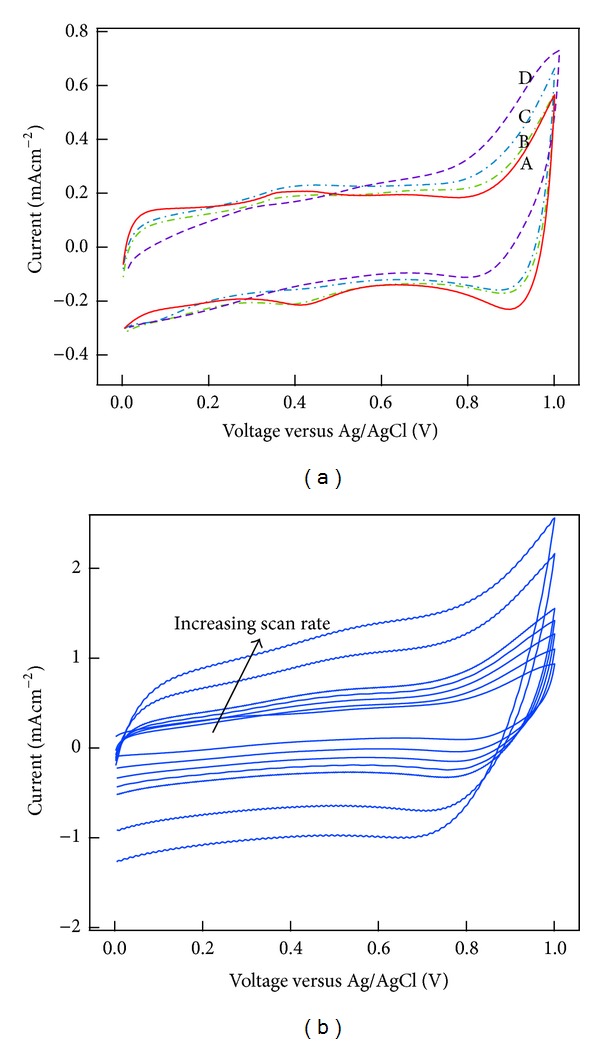
(a) Cyclic voltammograms obtained at a scan rate of 10 mVs^−1^ for untreated MWCNTs/MnO_2_ and wpCNTs/MnO_2_ under different plasma conditions ((A): untreated, (B): 75 W, 135 Pa, (C): 140 W, 135 Pa, (D): 10 W, 135 Pa). (b) Cyclic voltammograms of nanocomposite wpCNTs/MnO_2_ (10 W, 135 Pa) at different scan rates, 10, 20, 30, 40, 50, 100, and 150 mVs^−1^.

Highest specific capacitance (750 Fg^−1^) was obtained using plasma-treated CNTs with 10 W rf power and 135 Pa water pressure as the nanocomposite electrode. The increase in capacitance is related to the removal of amorphous carbon, soft etching, that is, less structural defects in comparison with other water plasma-treated carbon nanotubes, and the addition of oxygen functional groups [18]. Under these water-plasma conditions (10 W, 135 Pa), the existence of C=O (quinone) groups on the CNTs surface is especially high [18]. This type of groups promotes the adsorption of protons, which is in agreement with the nonperfect rectangular shape of the CV curves (i.e., a nontruly horizontal value of the current) [32] (see Figure 5(a)). As a result, the specific capacitance significantly increases due to the additional contribution of faradic currents (*I*_F_). Samples treated under other plasma conditions clearly show a redox peak in the potential region from 0.4 to 0.6 V that corresponds to reversible redox reactions between Mn^4+^ and Mn^3+^ (Figure 4(a)). With increasing scan rate, the specific capacitance of the wpCNTs/MnO_2_ (10 W, 135 Pa) composite decreases below that of the other samples. Since the shape of the CV curves becomes more rectangular at higher scan rates (Figure 5(b)), we assume that, for this sample, the *I*_F_ contribution to the total capacitance decreases due to slow Faradic processes taking place at the CNTs/MnO_2_ surface.

**Figure 6:**
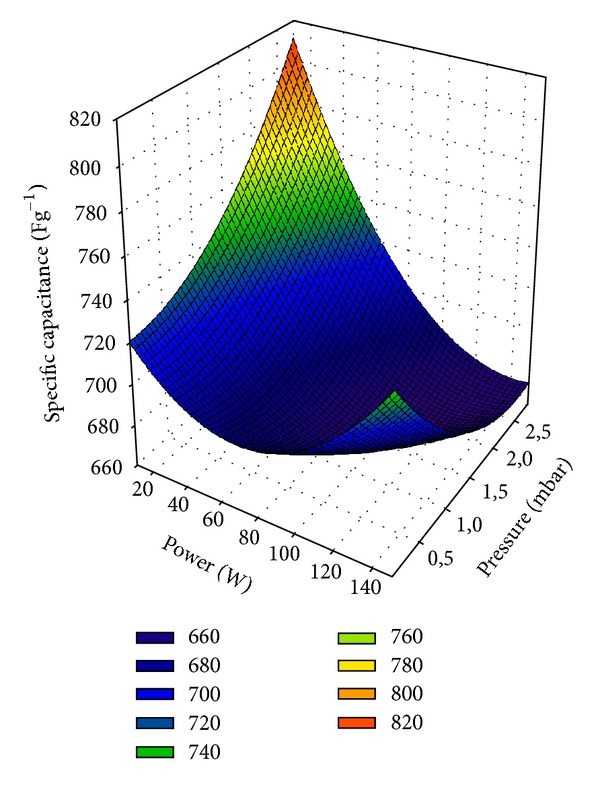
3-dimensional graph of the specific capacitance of water plasma-treated CNTs/MnO_2_ electrodes with respect to rf-power and water pressure.

Based on the experiments carried out, a second degree polynomial equation was adjusted for the specific capacitance (3). The polynomial model was found to be statistically significant (*P* < 0.05) explaining 76.6% of the samples variation with *α*_0_ = 730.935, *α*_1_ = −1.134, *α*_2_ = 4.723, *α*_3_ = 0.008, *α*_4_ = −0.415, and *α*_5_ = 11.365.  Figure 6 is a 3D graph obtained using these parameters. The interaction between plasma power and water pressure and their influence on the specific capacitance of wpCNTs/MnO_2_ composite electrodes is clearly shown. Optimum conditions of the plasma treatment that provide improved specific capacitance values of the composites are located at low plasma powers and high water pressures. 

Electrochemical impedance spectroscopy was performed to investigate the kinetics behaviour of hybrid electrodes. The internal components of the capacitor (e.g., current collectors, electrodes, dielectric material, and solution) contribute to the equivalent series resistance (ESR), which is above 65 ohm for all of the samples (see supplementary material, Figure  S3). The ESR of all the wpCNTs/MnO_2_ composites is lower than that of untreated CNTs/MnO_2_ composite (83 ohm) because of the removal of amorphous carbon and increased wettability. The Nyquist plot of the samples present an almost straight line parallel to the imaginary axis (see supplementary material, Figure  S3) that describes exactly polarized systems [1]. Deviation from this vertical line at low frequencies to smaller slopes corresponds to a higher contribution of the ionic diffusion resistance.

The cycling stability of nanocomposite electrodes was investigated applying galvanostatic charge/discharge cycles. A constant current density of 0.52 mA cm^−2^, in a potential window from 0 to 1 V, and of 1.75 mA cm^−2^, between 0.1 and 0.8 V, was applied during 2000 cycles. The specific capacitance was calculated from the discharge curve using (5) as follows:

(5) $$\begin{array}{c} C_{S}=\left(\frac{I}{\left(\Delta V/\Delta t\right)\cdot m}\right), \end{array}$$

where *C*_*s*_ is the specific capacitance in Fg^−1^, Δ*V* is the voltage difference during the discharge curve in V, *I* is the current in A, Δ*t* is the discharge time in s, and *m* has the same meaning and units as in (4).

**Figure 7:**
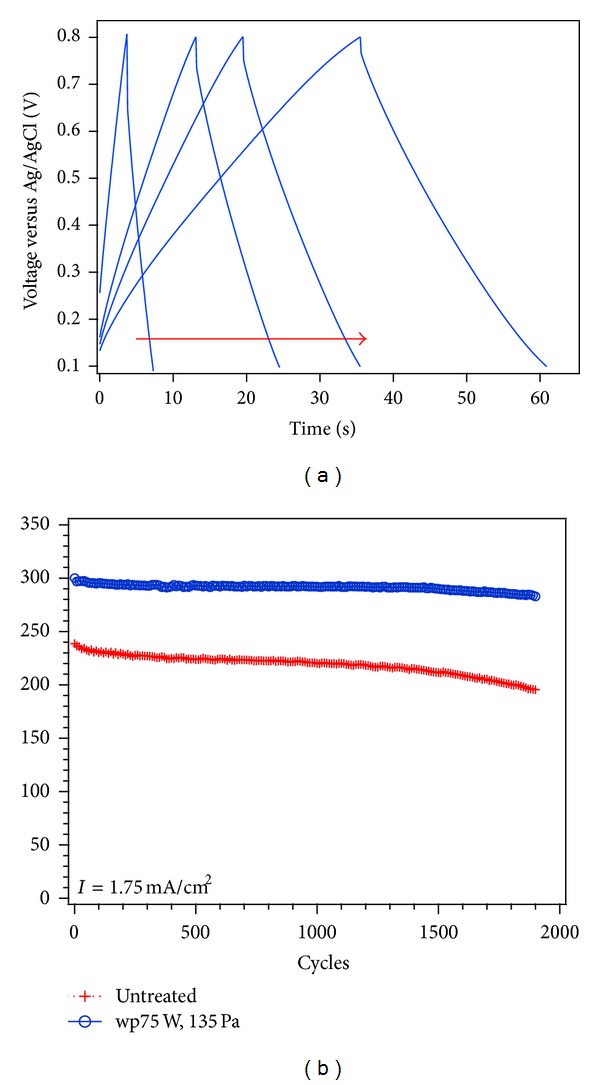
(a) Charge/discharge curves at different current densities from 1.75, 0.70, 0.52, and 0.35 mA cm^−2^, as indicated by the arrow, for wpCNTs/MnO_2_ (75 W, 135 Pa). (b) Galvanostatic charge/discharge cyclic stability in the 0.1–0.8 V potential.

Galvanostatic charge/discharge curves were measured applying various current densities from 1.75 to 0.35 mA cm^−2^.  Figure 7(a) shows charge/discharge curves of wpCNTs/MnO_2_ (75 W, 135 Pa) nanocomposite between 0.1 and 0.8 V. As with the EIS measurements, the ohmic drop of the curves indicates a high series resistance related to the contact between silicon substrate and nanotubes, current collectors, and solution resistance.  Figure 7(b) shows the cycling stability of nanocomposite electrodes in the voltage range of 0.1 to 0.8 V at a current density of 1.75 mA cm^−2^. It is evident that water plasma-treated CNTs/MnO_2_ exhibits both a higher capacitance and capacitance retention in comparison with untreated CNTs/MnO_2_. On the other hand, charge/discharge cycling of untreated CNTs/MnO_2_ nanocomposite electrode in the voltage window of 0 to 1 V (see supplementary material, Figure S2) gives higher capacitance than wpCNTs/MnO_2_ samples. However, it only lasts up to 700 cycles, while, in the case of wpCNTs/MnO_2_ samples, a continuous fading in the capacitance was observed. The reason for this poor cyclability in both composites with untreated and plasma-treated CNTs is related to irreversible reduction/oxidation reactions of Mn^4+^ to Mn^2+^ and Mn^7+^ in the voltage region around 0 V and 1 V, respectively. Additionally, oxygen evolution reaction starts to take place around 0.8 V, as observed in Figure 5(a), which could also affect the reversibility of redox reactions [10]. These irreversible redox reactions cause the dissolution of manganese in the electrolyte and fading of the capacitance [33] (see Figure S2). 

From the above results, we assume that some processes occur during cycling in the potential regions around 0 V and 1 V, namely, dissolution of MnO_2_ nanoflowers, coalescence, and upward drag of the oxide forming a thick layer of material on top of the CNTs, and morphological modification of the nanoflowers to a needle-like structure that corresponds to hausmannite Mn_3_O_4_. The dense and thick layer of Mn_3_O_4_ on the CNTs mat generated during the cycling process appears to be the reason for the capacitance fading. Furthermore, the presence of Mn_3_O_4_ proved by Raman spectroscopy and XPS is known to significantly decrease the reversibility of the system [21]. SEM images and cyclability experiments reveal that highly porous CNTs/MnO_2_ composite electrodes, with good electronic conduction and, most importantly, separated MnO_2_ nanoflowers, and/or layer-like structure are the desired conditions for a suitable capacitive behaviour. 

## 4. Conclusions

Water plasma treatment performed on CNTs removes amorphous carbon and introduces several oxygen functional groups on the CNTs surface. MnO_2_/CNTs nanocomposite electrodes were obtained after galvanostatic deposition of MnO_2_ on untreated and water plasma-treated CNTs. SEM images confirm the removal of amorphous carbon and reveal nanoflower-structured MnO_2_ deposited on the CNTs. Raman spectroscopy and XPS results indicate that electrochemical cycling in the potential range from 0-1 V converts MnO_2_ to Mn_3_O_4_, which corresponds to a change in the metal oxide morphology observed with SEM. On the other hand, a voltage window between 0.1–0.8 V does not change the oxidation state of MnO_2_ and only induces a morphological change from nanoflower to layer-like structure of the oxide. High specific capacitance values were achieved under optimum water-plasma conditions, low rf plasma powers and high water pressures. A high specific capacitance of 750 Fg^−1^ was obtained for wpCNTs/MnO_2_ (10 W, 135 Pa) nanocomposite electrode.

## Supplementary Material

This supplementary material provides additional information about Box-Wilson experimental design (figure S1), charge/discharge curves and cycling between 0 and 1 V (figure S2), Nyquist plot of different samples (figure S3), and a table regarding the O1s peak in the XPS spectra of samples with MnO_2_ before and after cycling (table S4).

## Figures and Tables

**Table 1:** Specific capacitance values of untreated and water plasma-treated CNTs/MnO_2_ composite at a scan rate of 10 mVs^−1^.

Sample	*C*_*s*_ (Fg^−1^)	Sample	*C*_*s*_ (Fg^−1^)
Untreated MWCNTs	678	75 W, 135 Pa	669
10 W, 135 Pa	750	75 W, 260 Pa	696
29 W, 50 Pa	686	121 W, 50 Pa	669
29 W, 220 Pa	746	121 W, 220 Pa	665
75 W, 10 Pa	713	140 W, 135 Pa	694
