# Supplementary material for: Water Plasma Functionalized CNTs/MnO2 Composites for Supercapacitors
Source: ScientificWorldJournal. 2013 Nov 19;2013:832581. doi: 10.1155/2013/832581 (PMC3853549; doi:10.1155/2013/832581)
Supplement: Supplementary file 1 — This supplementary material provides additional information about Box-Wilson experimental design (figure S1), charge/discharge curves and cycling between 0 and 1 V (figure S2), Nyquist plot of different samples (figure S3), and a table regarding the O1s peak in the XPS spectra of samples with MnO2 before and after cycling (table S4). [file 832581.f1.pdf]

Supplementary information

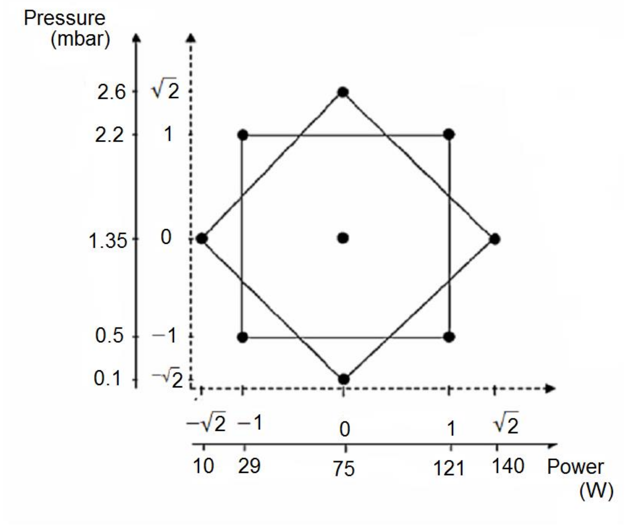

Fig. S1. Box-Wilson experimental design samples distribution for two main parameters (RF power and water pressure).

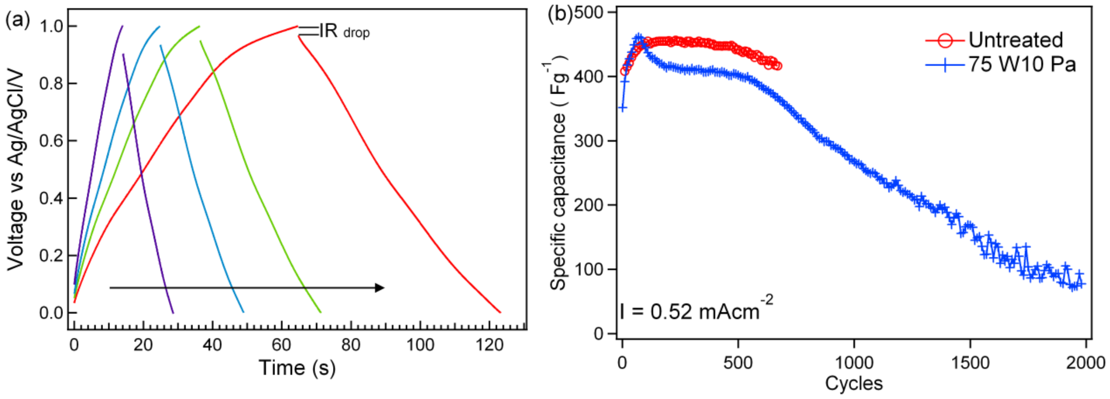

Fig. S2. (a) Charge/discharge curves obtained at different current densities from 0.1, 0.2, 0.3, 0.4 $mAcm^{-2}$, as indicated by the arrow for wpCNTs/MnO_2 (10 W, 135 Pa) and (b) charge/discharge cyclic stability between 0 and 1 V for untreated and water plasma treated CNTs/MnO_2 electrodes.

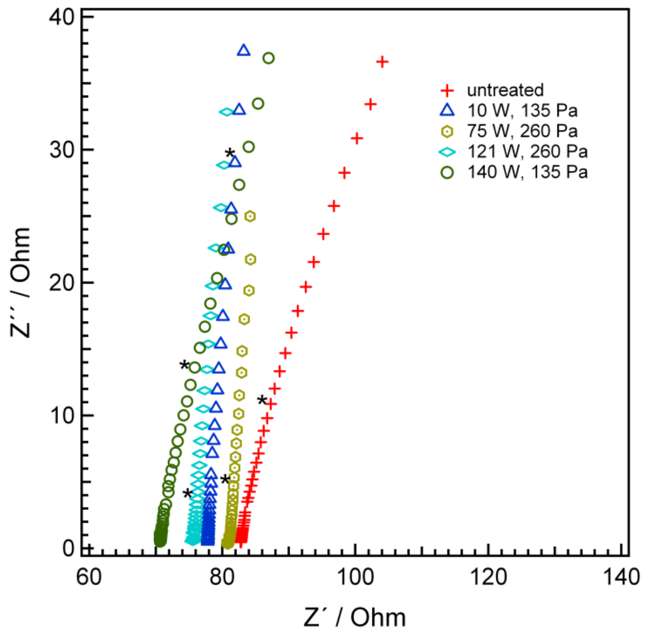

Fig. S3 Nyquist plot of untreated and different wpCNTs/MnO_2 samples. Asterisks correspond to a frequency of 9.32 Hz.

Table S4. Peak position and percentage area of different bonds obtained from XPS O1s spectra .

Sample		(BE) (eV)	% area
wpCNTs/MnO_2 before cycling	Mn-O-Mn	529.44	33.79
	Mn-O-OH	530.88	24.36
	H-O-H	532.39	13.89
	Chemisorbed oxygen	535.26	27.96
wpCNTs/MnO_2 (0 – 1 V) after 2000 cycles	Mn-O-Mn	529.29	20.88
	Mn-O-OH	530.71	13.69
	H-O-H	523.36	7.68
	Chemisorbed oxygen	535.07	57.75
wpCNTs/MnO_2 (0.1 – 0.8 V) after 2000 cycles	Mn-O-Mn	529.31	23.17
	Mn-O-OH	530.84	17.37
	H-O-H	532.55	3.7
	Chemisorbed oxygen	534.91	55.75
